# Clinical and biochemical correlates of hypogonadism in men with type 2 diabetes mellitus

**DOI:** 10.11604/pamj.2021.38.292.25719

**Published:** 2021-03-19

**Authors:** Ezekiel Musa, Jibril Mohammed El-Bashir, Fatima Sani-Bello, Adamu Girei Bakari

**Affiliations:** 1 Department of Internal Medicine, Kaduna State University, Kaduna, Kaduna State, Nigeria,; 2 Division of Endocrinology, Department of Medicine, University of Cape Town, Cape Town, South Africa,; 3 Department of Chemical Pathology, Ahmadu Bello University, Zaria, Kaduna State, Nigeria,; 4 Department of Medicine, Ahmadu Bello University, Zaria, Kaduna State, Nigeria

**Keywords:** Type 2 diabetes mellitus, hypogonadism, total testosterone, testicular volume

## Abstract

**Introduction:**

there is an association between hypogonadism and obesity, chronic hyperglycaemia, and ageing in men with type 2 diabetes mellitus (T2DM). T2DM is known to be associated with low testosterone. There is a paucity of data on the risk factors of hypogonadism in Nigerian men with T2DM. The objective of this study was to determine the clinical and biochemical correlates of hypogonadism and clinical predictors of low total testosterone levels in men with T2DM.

**Methods:**

this was a cross-sectional study consisting of 358 men with T2DM and 179 non-diabetic men (controls). Structured Androgen Deficiency in the Ageing Male questionnaire was administered. Clinical and biochemical parameters were measured. Free testosterone was calculated from albumin, SHBG and total testosterone using Vermeulen´s method. Hypogonadism was defined as fasting TT as < 8 nmol/L with or without symptoms or TT of 8-12 nmol/L with symptoms of androgen deficiency. Low testosterone was defined as serum total testosterone levels ≤ 12 nmol/L.

**Results:**

the mean (±SD) total testosterone of men with T2DM and controls were 8.79±3.35 nmol/L and 15.41±3.79 nmol/L respectively (p < 0.001). The risk of hypogonadism was associated with central obesity (Odds ratio [OR] 2.24, 95% confidence interval [CI] 0.38-13.07), systolic hypertension (OR 3.93, 95% CI 0.67-23.10), hyperglycaemia (OR 2.48, 95% CI 0.37-16.46) and hypercholesterolaemia (OR 2.50, 95% CI 0.43-14.61). In a multivariable regression analysis, there was a significant negative correlation between total testosterone and triglycerides (r -1.85, 95% CI -3.58 - 0.12, P = 0.04) and HDL cholesterol (r -1.25, 95% CI -5.95-3.45, P = 0.02).

**Conclusion:**

this study shows that in men with T2DM, triglycerides and HDL cholesterol are independent correlates of hypogonadism but not central adiposity, systolic blood pressure and glycaemia. Further large prospective studies are recommended.

## Introduction

Hypogonadism is a clinical syndrome comprising both symptoms, with or without signs, combined with biochemical evidence of testosterone deficiency [1,2]. In cross-sectional studies, between 20-80.4% of men with T2DM have hypogonadism [3-5]. Type 2 diabetes mellitus has been reported to be associated with low testosterone [3, 6-9]. Low testosterone levels are reportedly associated with a high risk of developing T2DM in men, a sequel to its insulin-resistance effect [10, 11]. Some of the clinical features of symptomatic hypogonadism are erectile dysfunction, loss of libido, depression, irritability, fatigue, anaemia, decreased intellectual activity, sleep disturbance, increased abdominal fat, decreased body hair and bone mineral density and lean body mass [2]. Erectile dysfunction, a commonly documented hypogonadism feature, was three times more common in men with T2DM than those without DM [12]. Furthermore, another study found that loss of libido and erectile dysfunction were the prominently reported symptoms by most type 2 diabetic men [9].

Cross-sectional studies have reported a significant relationship between body mass index (BMI), waist circumference and testosterone levels [7, 9, 13]. In South Africa, Paruk *et al*. found a significant relationship between ageing and BMI as well as an inverse relationship between total testosterone and BMI, waist circumference and metabolic syndrome [9]. Similarly, Kapoor *et al*. reported a significant negative correlation between testosterone levels and BMI and waist circumference in men with T2DM [7]. Meanwhile, another study found BMI was inversely correlated with both free and total testosterone and a strong correlation between equilibrium dialysis-measured free testosterone and calculated free testosterone but not with SHBG [3]. Furthermore, Kemp *et al*. reported waist circumference and known cardiovascular disease as the significant factors associated with low total testosterone [13]. Ugwu *et al*. reported older age and central obesity as the predictors of hypogonadism among type 2 diabetic men [14].

There is currently a paucity of data on the risk factors of hypogonadism in Nigerian men with T2DM. Availability of relevant clinical and biochemical markers that will signal the early screening and diagnosis of hypogonadism in men with T2DM is timely. This will perhaps herald the appropriate care and improvement of the quality of life of men with T2DM. This study aimed to determine the clinical and biochemical correlates of hypogonadism in men with T2DM as well as the clinical predictors of low testosterone in men with T2DM.

## Methods

### Study design and setting

This was a cross-sectional study undertaken at the endocrine clinic of the Ahmadu Bello University Teaching Hospital (ABUTH), Zaria, from October 2015 to June 2016.

### Study population

The study consisted of 358 male patients with T2DM and 179 non-diabetic men (controls). Men with T2DM (age 21 years and above) were recruited consecutively by convenience sampling as they fulfilled all the inclusion criteria. One hundred and seventy-nine healthy non-diabetic men were recruited consecutively. Those with chronic liver disease, chronic kidney disease, panhypopituitarism, HIV, type 1 diabetes mellitus, suspected prostate or testicular cancer, history of previous or present treatment of hypogonadism with testosterone and anti-androgen or related drugs, hospital admission, age less than 21 years, and refusal to participate in the study were excluded. Additionally, 43 men each with T2DM and controls were randomly selected in a case-control fashion using a computer from the cohorts of 358 men with T2DM and 179 controls, respectively, for sub-analysis.

### Data collection

A structured Androgen Deficiency in the Ageing Male (ADAM) questionnaire was administered to obtain low testosterone symptoms. Anthropometric measurements were obtained. Body mass index (BMI) was calculated as weight in kilogram (kg) divided by height in meters squared (m^2^). Weight was measured in kg with the subjects standing steadily on a scale, and height was measured in meters using a stadiometer. According to the World Health Organization (WHO) protocol, Waist circumference was measured at the midpoint between the iliac crest and the costal margin (lower rib). Meanwhile, hip circumference was measured at the widest portion of the buttocks with the tape parallel to the floor [15, 16]. The BMI classification was based on the WHO definition for obesity, while waist circumference cut-off of ≥ 94 cm defined abdominal obesity based on the International Diabetes Federation (IDF) criteria [17, 18]. Blood pressure (BP) was measured by a sphygmomanometer, and participants´ BP was considered uncontrolled if their target BP was ≥ 140/90 mmHg, according to the American Diabetes Association (ADA) recommendations [19]. A Prader orchidometer was used to measure the testicular volume of participants [20].

The laboratory biochemical tests performed on all participants included glycated haemoglobin (HbA1c), fasting plasma glucose (FPG), fasting serum total testosterone (TT), sex hormone-binding globulin (SHBG), albumin and lipid profile. Free testosterone was calculated from albumin, SHBG and total testosterone using Vermeulen´s method [21]. The Glycated haemoglobin and FPG targets were based on ADA recommendations [22]. Fasting venous blood samples (10 ml) were collected in the morning between 8: 00 AM and 10: AM into plain tubes and allowed to clot within 30 minutes. Samples were centrifuged for 10 minutes, and sera were collected into sample bottles and frozen at -20^o^C for future analysis. The sera samples were used for the analysis of total testosterone, SHBG, albumin and lipid profile. Separate 4 ml of blood was collected into an ethylenediaminetetraacetic acid (EDTA) bottle for HbA1c assay.

Glycated haemoglobin was measured using the Clover A1C (Infopia Co. Ltd, Gyeonggi-do, South Korea), plasma glucose was measured using the oxidase-peroxidase method, serum albumin was measured by modified bromocresol green colorimetric method using kit acquired from Randox laboratory limited, Crumlin, United Kingdom. Lipid profile was measured using reagents acquired from Randox laboratory limited, Crumlin, United Kingdom. SHBG was measured by enzyme immunoassay technique using kits acquired from Diagnostic Automation Inc, California, USA, and serum total testosterone was measured by the enzyme immunoassay technique using Testosterone AccuBind ELISA kits from Monobind Inc, California, USA.

### Definitions

Hypogonadism was defined as total testosterone levels < 8 nmol/L with or without symptoms or total testosterone levels of 8-12 nmol/L with the presence of symptoms. Low testosterone was defined as serum total testosterone levels ≤ 12 nmol/L.

### Statistical analysis

Data were analysed using Statistical Package for Social Sciences (SPSS) version 21 after validation. Quantitative variables such as BMI, lipid profile and testosterone levels were presented as means and standard deviations. The Student's t-test was used to determine the difference in means of clinical and biochemical parameters between type 2 diabetic men and controls. Chi-Square and regression analysis were used to analyse associations between hypogonadism and clinical and biochemical parameters, and univariable and multivariable correlations between clinical and biochemical parameters and low testosterone, respectively. Logistic regression analysis was used to determine the clinical predictors of low testosterone levels among men with T2DM. P values < 0.05 were considered statistically significant.

### Ethical considerations

All participants gave written informed consent. Ethical clearance for the study was obtained from ABUTH ethical committee (ABUTHZ/HREC/NP18/2015), and confidentiality was observed throughout the study.

## Results

### General characteristics of the study population

A total of 358 men with T2DM and 179 controls participated in the study. The mean (±SD) age for the men with T2DM was 46.34±5.66 years, while that of the controls was 44.09 ± 12.39 years (p = 0.57). In our study, 177 (49.4%) of the men with T2DM had a duration of diabetes ranging 0-5 years, while 91 (25.4%), 55 (15.4%), 13 (3.6%), and 22 (6.2%) had diabetes for a duration of 6-10, 11-15, 16-20 and more than 20 years respectively (Table 1). Among the six categories of medications men with T2DM were taken, majority (34.6%) were on a metformin-glimepiride combination. Those who received metformin alone and metformin and insulin combinations were 55 (15.4%) and 52 (14.5%), respectively (Table 1). Two hundred and sixty-nine (75%) of the men with T2DM were hypertensive, with the majority (73.2%) on bendrofluazide, while 43.3% were taking lisinopril. The mean (±SD) systolic blood pressure (SBP) for men with T2DM and controls was 143.36±25.64 mmHg and 128.70±22.59 mmHg, respectively. There was a statistically significant difference between men's mean SBP with T2DM and the controls (p < 0.001, Table 1). Also, the mean (±SD) diastolic blood pressure (DBP) of men with T2DM was statistically higher than the controls (82.54 ± 13.05 mmHg versus 79.82±14.05 mmHg, p = 0.03, Table 1). Abdominal obesity was found in 43.6% of men with T2DM, and the mean waist circumference of type 2 diabetic men was higher than the controls (92.40±11.96 cm versus 79.49±9.84 cm, p < 0.001) (Table 1). The mean (±SD) testicular volume of men with T2DM and controls was 20.01±4.15 ml and 21.37±3.54 ml, respectively. The mean testicular volume of the controls was higher than that of the men with T2DM, which was statistically significant (p < 0.001, Table 1). Three hundred and seven (85.8%) of the men with T2DM did not achieve target metabolic control using glycated haemoglobin. The mean (±SD) glycated haemoglobin was significantly higher in men with T2DM compared to controls (p < 0.001) (Table 1). There was a significant difference between type 2 diabetic men and controls for their mean total cholesterol (p < 0.001), LDL cholesterol (< 0.001), and triglycerides (< 0.001) (Table 1).

**Table 1:** clinical and biochemical features of men with T2DM and controls

	T2DM	Controls	
	n = 358	n = 179	Significance
	Mean±SD	Mean±SD	P-value
**Age** (years)	46.34±5.66	44.09 ± 12.39	0.57
**Clinical parameters:**			
SBP (mmHg)	143.36±25.64	128.70 ± 22.59	<0.001
DBP (mmHg)	82.54±13.05	79.82 ± 14.05	0.03
WC (cm)	92.40 ± 11.96	79.49 ± 9.84	<0.001
BMI (m/kg2)	25.26 ± 4.45	24.31 ± 3.79	0.01
Testicular volume (ml)	20.01 ± 4.15	21.37 ± 3.54	<0.001
**Biochemical parameters:**			
FPG (mmol/L)	8.30 ± 4.77	4.43 ± 1.79	<0.001
HbA1c (%)	9.13 ± 2.12	5.07 ± 0.74	<0.001
Total cholesterol (mmol/L)	4.28 ± 0.92	3.98 ± 0.70	<0.001
LDL cholesterol (mmol/L)	2.63 ± 0.74	2.37 ± 0.56	<0.001
HDL cholesterol (mmol/L)	1.04 ± 0.33	1.10 ± 0.34	0.06
Triglycerides (mmol/L)	1.32 ± 0.49	1.11 ± 0.37	<0.001
Total testosterone (nmol/L)	8.79 ± 3.35	15.41 ± 3.79	<0.001
SHBG (nmol/L)	85.59 ± 63.96	95.51 ± 76.29	0.14
Albumin (mmol/L)	39.68 ± 3.26	41.53 ± 3.42	<0.001
Calculated free testosterone (nmol/L)	0.11 ± 0.07	0.22 ± 0.16	<0.001
**Duration of T2DM (years) n (%):**			
0-5	177 (49.4)		
6-10	91 (25.4%)		
11-15	55 (15.4%)		
16-20	13 (3.6%)		
> 20	22 (6.2%)		
**Medications n (%):**			
Metformin alone	55 (15.4%)		
Metformin + glimepiride	124 (34.6%)		
Metformin + glibenclamide	78 (21.8%)		
Metformin + glimepiride + insulin	8 (2.2%)		
Metformin + glibenclamide + insulin	6 (1.7%)		
Metformin + insulin	52 (14.5%)		
Others	35 (9.8%)		

P <0.05 is statistically significant. BMI: body mass index, DBP: diastolic blood pressure, FBG: fasting plasma glucose, HbA1c: glycated haemoglobin, HDL: high density lipoprotein, LDL: low density lipoprotein, SHBG: sex hormone-binding globulin, SBP: systolic blood pressure, WC: waist circumference. Please note that BMI, FPG, HbA1c, total testosterone and WC of men with T2DM and controls have been published already (Musa et al 2021) [5].

### Association of clinical and biochemical parameters with hypogonadism in men with T2DM

A sub-group analysis of 43 men with T2DM was performed to assess the risk factors of hypogonadism. All obese type 2 diabetic men had hypogonadism, with grade 1 obesity accounting for all the cases. None of the hypogonadal men with T2DM had either grade 2 or morbid obesity. Among the type 2 diabetic men who had abdominal obesity, 89.5% had hypogonadism, and 79.2% of the non-obese men with T2DM had hypogonadism. The odds of developing hypogonadism among type 2 diabetic men with abdominal obesity was twice that of the non-obese men, but this did not reach statistical significance (X^2^ = 0.83, p = 0.36, OR = 2.24, CI = 0.38 - 13.07, Table 2).

**Table 2:** association of clinical and biochemical parameters with hypogonadism in men with T2DM

Characteristics	Hypogonadism	Eugonadism	Significance
	Frequency (%)	Frequency (%)	P-Values
**BMI**			0.64
≥ 30 kg/m2	5 (100)	0 (0)	
< 30 kg/m2	31 (81.6)	7 (18.4)	
**Obesity by WC**			0.36
≥ 94 cm	17 (89.5)	2 (10.5)	
< 94 cm	19 (79.2)	5 (20.8)	
**Obesity by WHR**			0.66
≥ 0.90	35 (83.3)	7 (16.7)	
< 0.90	1 (100)	0 (0)	
**SBP**			0.11
≥ 140 mmHg	22 (91.7)	2 (8.3)	
< 140 mmHg	14 (73.7)	5 (26.3)	
**DBP**			0.92
≥ 90 mmHg	12(100)	0 (0)	
< 90 mmHg	24(77.4)	7 (22.6)	
**FPG**			0.58
>7.2 mmol/L	18 (90)	2 (10)	
4.4 - 7.2 mmol/L	14 (77.8)	4 (22.2)	
< 4.4 mmol/L	4 (80)	1 (20)	
**HbA1c**			0.34
≥7%	31(86.1)	5 (13.9)	
< 7 %	5(71.4)	2 (28.6)	
**T C**			0.46
≥ 5.2 mmol/L	6 (75)	2(25)	
< 5.2 mmol/L	30 (85.7)	5(14.3)	
**LDL cholesterol**			0.30
≥ 2.6 mmol/L	18 (78.3)	5 (21.7)	
< 2.6 mmol/L	18 (90)	2(10)	
**HDL cholesterol**			0.61
< 1.1 mmol/L	14 (87.5)	2(12.5)	
≥ 1.1 mmol/L	22 (81.5)	5(18.5)	
**Triglycerides**			0.17
≥ 1.7 mmol/L	8 (100)	0(0)	
< 1.7 mmol/L	28 (80)	7 (20)	

BMI: body mass index, DBP: diastolic blood pressure, FPG: fasting plasma glucose, HbA1c: glycated haemoglobin, HDL: high density lipoprotein, LDL: low density lipoprotein, SBP: systolic blood pressure, TC: total cholesterol, WC: waist circumference, WHR: waist-hip ratio.

Twenty-two (91.7%) of type 2 diabetic men with above-target systolic blood pressure (SBP) had hypogonadism, while 73.7% of those with SBP within target had hypogonadism as well. There was about a four-fold likelihood of developing hypogonadism among type 2 diabetic men with elevated SBP compared to normal SBP (X^2^ = 2.52, p = 0.11, OR = 3.93, CI = 0.67- 23.10, Table 2). All men with T2DM who had high diastolic blood pressure (DBP) were hypogonadal compared to 77.4% of those with normal DBP (X^2^ = 0.01, p = 0.92, OR = 1.10, CI = 0.18-6.56, Table 2). There was a higher frequency of hypogonadism (90%) among type 2 diabetic men with poor glycaemic control compared to those who achieved target fasting plasma glucose (FPG) using ADA criteria. Thirty-one (86.1%) of those who did not achieve target glycated haemoglobin had a higher prevalence of hypogonadism than those whose glycated haemoglobin was well-controlled (71.4%). The risk of having hypogonadism among men with T2DM with poor glycated haemoglobin was about 2.5 times more than with well-controlled glycated haemoglobin, but a statistically significant association between glycated haemoglobin and hypogonadism was not achieved (X^2^ =0.93, p=0.34, OR=2.48, CI = 0.37- 16.46, Table 2). Among the men with T2DM who had elevated total cholesterol, 75% had hypogonadism. Meanwhile, 85.7% of those who had normal total cholesterol were hypogonadal. There was no statistically significant association between total cholesterol level and hypogonadism (X^2^ = 0.55, p = 0.46, OR = 2.00, CI = 0.31-12.84, Table 2). The odds of having hypogonadism were 2.5 times higher in men with T2DM who had elevated LDL cholesterol as opposed to having normal LDL cholesterol (X^2^=1.08, p=0.30, OR=2.50, CI=0.43-14.61, Table 2).

### Univariable and multivariable regression analyses of correlates of hypogonadism in men with T2DM

In a multivariable regression, there was a significant negative correlation between total testosterone and triglycerides (r -1.85, 95% CI -3.58-0.12, p = 0.04) and HDL cholesterol (r -1.25, 95% CI -5.95-3.45, p = 0.02) (Table 3). However, a univariable regression showed a significant positive correlation between total testosterone and LDL cholesterol (r 0.38, 95% CI 0.38-2.92, p = 0.01).

**Table 3:** univariable and multivariable correlates of hypogonadism in men with T2DM

	Univariable analysis		Multivariable analysis	
	Coefficient of Correlation (r) (95% CI)	P-value	Coefficient of Correlation (r) (95% CI)	P-value
**FPG**	0.07 (-0.30 - 0.18)	0.64	-0.08 (-0.53 - 0.37)	0.72
**HbA1C**	0.01 (-0.44 - 0.43)	0.97	0.33 (-0.50- 1.15)	0.43
**TC**	0.28 (-0.09 - 1.95)	0.07	1.79 (0.01 - 3.56)	0.83
**HDL**	0.18 (-1.14 - 4.14)	0.26	-1.25 (-5.95 - 3.45)	0.02*
**LDL**	0.38 (0.38 - 2.92)	0.01*	1.62 (-0.10 - 3.34)	0.06
**TG**	0.25 (-2.45 - 0.27)	0.11	-1.85 (-3.58 - 0.12)	0.04*
**SBP**	0.05 (-0.03 - 0.04)	0.77	-0.07 (-0.07 - 0.05)	0.82
**DBP**	0.03 (-0.06 - 0.07)	0.84	0.04 (-0.08 - 0.15)	0.52
**BMI**	0.09 (-0.31 - 0.17)	0.57	-0.26 (-0.70 - 0.19)	0.25
**WC**	0.01 (-0.09 - 0.09)	0.96	0.10 (-0.10 -0.29)	0.33
**WHR**	0.12 (-24.14 - 11.03)	0.46	-4.67 (-30.47-21.14)	0.72

Note: *p<0.05 is statistically significant. BMI: body mass index, DBP: diastolic blood pressure, FPG: fasting plasma glucose, HbA1c: glycated haemoglobin, HDL: high density lipoprotein, LDL: low density lipoprotein, SBP: systolic blood pressure, WC: waist circumference, WHR: waist-hip ratio.

### Logistic regression analysis of the clinical predictors of low total testosterone in men with T2DM with hypogonadism

In a logistic regression analysis using total testosterone as the dependent variable and average testicular volume, BMI, erectile dysfunction, decreased libido, duration of diabetes and waist circumference as independent variables, the clinical variables that had a higher likelihood of predicting low total testosterone levels in men with T2DM were erectile dysfunction (OR 6.00, 95% CI 0.97-37.12, p = 0.05) and a decreased libido (OR 4.67, 95% CI 0.86-25.31, p = 0.07) (Table 4).

**Table 4:** logistic regression analysis of the clinical correlates of low total testosterone in men with T2DM with hypogonadism

	P-values	Odd Ratio	95% CI	
			Lowerbound	Upperbound
Average testicular volume	0.10	0.00	0.00	0.00
BMI	0.37	0.61	0.20	1.80
WC	0.37	2.24	0.38	13.07
ED	0.05	6.00	0.97	37.12
Decreased libido	0.07	4.67	0.86	25.31
Duration of diabetes mellitus	0.20	0.45	0.13	1.52

BMI: body mass index, ED: erectile dysfunction, WC: waistcircumference.

## Discussion

This study found a significantly lower total and calculated free testosterone in men with T2DM than non-diabetic controls. Asare-Anane *et al*. and Onung *et al*. found similar findings in Accra, Ghana and Lagos, Nigeria, respectively [6, 23]. Furthermore, Paruk *et al*. in South Africa also found lower total and free testosterone levels in type 2 diabetic men than in the controls. Two Meta-analyses by Ding *et al*. and Corona *et al*. which included 20 cross-sectional studies (850 diabetic men and 2000 non-diabetic controls) and 28 cross-sectional studies (1,822 men with diabetes and 10,009 non-diabetic controls), respectively, found consistent lower total testosterone levels in men with diabetes compared with non-diabetic controls [24, 25]. The association between low total testosterone and diabetes has been previously attributed to low SHBG observed commonly in T2DM [26]. However, in our study, calculated free testosterone was significantly lower in men with T2DM than non-diabetic controls, suggesting that other factors may play a role in lowering testosterone levels and not SHBG alone.

Despite the higher risk of hypogonadism among type 2 diabetic men with poor glycaemic control, the association between poor glycaemic control and hypogonadism did not reach statistical significance but was clinically significant. This finding is consistent with a report from a Nigerian study [8]. Some studies reported a significant association between hypogonadism and FPG and glycated haemoglobin [6, 7]. Prolonged hyperglycaemia impairs the synthesis and secretion of testosterone, resulting in low testosterone levels. The high proportion of type 2 diabetic men with uncontrolled and well-controlled glycated haemoglobin may explain the apparent insignificant statistical association between glycaemia and hypogonadism. This study did not show a statistically significant association between total cholesterol and hypogonadism. It was similar to findings from Nigerian and United Kingdom studies [4, 8]. A study reported a significant association with low total testosterone [27].

Furthermore, this study showed no statistically significant association between LDL cholesterol and hypogonadism, despite the high mean LDL cholesterol in hypogonadal subjects. This finding is supported by Mirzaei *et al*.’s study, which found no correlation between total testosterone and lipid profile reported [28]. Conversely, Wickramatilake and colleagues showed a significant association between LDL cholesterol and hypogonadism [27]. The association observed between hypogonadism and both systolic and diastolic blood pressure was not significant in this study. These findings are consistent with those reported in other studies [4, 29]. In contrast, some studies reported an observed association between hypogonadism and blood pressure [7, 30]. There was an observed clinically but not a statistically significant association between hypogonadism and abdominal obesity using waist circumference despite a higher frequency of abdominal obesity in hypogonadal type 2 diabetic men. Zheng *et al*. also reported a lack of significant association between hypogonadism and abdominal obesity but the opposite finding was reported in a study by Laaksonen *et al*. [31, 32]. This study found no association between hypogonadism and global obesity similar to prior studies [8, 33]. Observations by some researchers showed a significant association between obesity (using BMI) and hypogonadism [13, 34]. The lack of a statistically significant association between obesity and hypogonadism despite the higher odds of developing hypogonadism in obese type 2 diabetic men compared to non-obese could be attributed to a considerably high proportion of hypogonadism in the non-obese men.

There was a significant negative correlation between total testosterone and triglycerides and HDL cholesterol in multivariable analysis with a linear decrease in the total testosterone levels as these variables increased. These findings were consistent with other researchers reports [34, 35] but contradicted the findings by Asare-Anane *et al*. and Chang *et al*. [6, 36]. These indicate that male patients with T2DM who have dyslipidaemia may often exhibit hypogonadism. Low testosterone is associated with increased adiposity, T2DM, increased inflammation, hyperlipidaemia [37]. The findings in this study show no significant association between total testosterone and anthropometric measurements and FPG as opposed to the findings by Kapoor *et al*. and Asare-Anane *et al*. [6, 7].

Erectile dysfunction and decreased libido showed a higher likelihood of predicting low total testosterone levels, with the odds approaching statistical significance. Meanwhile, waist circumference, testicular volume, duration of diabetes and BMI were not found to increase the likelihood of predicting low total testosterone. The ADAM questionnaire, which has a sensitivity of 88%, established that erectile dysfunction and loss of libido are predictors of low testosterone [38]. In contrast to the findings in this study, Mahmoud *et al*. found testicular volume was a predictor of low testosterone [39] while Travison *et al*. reported that obesity using WC and BMI and duration of diabetes as predictors of low total testosterone [40]. The much lower cut-off for defining abdominal obesity in sub-Saharan African men, according to IDF, may account for the seeming lack of statistical significance despite the observed higher odds of having low testosterone with abdominal obesity. Additionally, the exceptionally high proportion of both hypogonadal and eugonadal men who gave positive responses to erectile dysfunction and loss of libido could have attenuated any inherent significance.

This study, being hospital-based, might not have given an actual reflection of hypogonadism in men with T2DM. Also, considering its cross-sectional nature, findings are mainly associations, therefore, a well-designed prospective study to investigate the clinical and biochemical correlates of hypogonadism in male patients with T2DM is recommended.

## Conclusion

This study shows that triglycerides and HDL cholesterol are independent correlates of hypogonadism in men with T2DM. There was no association between hypogonadism and abdominal obesity, systolic hypertension, and hyperglycaemia in men with T2DM. Erectile dysfunction and decreased libido are the likely clinically important predictors of low testosterone levels in type 2 diabetic men with hypogonadism.

### What is known about this topic

- An association between hypogonadism and obesity and chronic hyperglycaemia in men with T2DM exists;
- T2DM is known to be associated with low testosterone in male patients.

### What this study adds

- This study found a lower testicular volume in men with T2DM than non-diabetic men; to the best of our knowledge, this is the first study in Africa to utilise a Prader orchidometer in assessing testicular volume in T2DM;
- This is also the first study to determine calculated free testosterone using Vermeulen´s equation with approximate precision to equilibrium dialysis (goal standard) for measuring serum free testosterone; and our research found lower levels of free testosterone in men with T2DM compared to non-diabetics;
- Our findings suggest that erectile dysfunction and decreased libido may be clinically important in assessing hypogonadism in men with T2DM.
